# Elucidating SNP-based genetic diversity and population structure of advanced breeding lines of bread wheat (*Triticum aestivum* L*.*)

**DOI:** 10.7717/peerj.11593

**Published:** 2021-06-22

**Authors:** Vipin Tomar, Guriqbal Singh Dhillon, Daljit Singh, Ravi Prakash Singh, Jesse Poland, Arun Kumar Joshi, Budhi Sagar Tiwari, Uttam Kumar

**Affiliations:** 1 Borlaug Institute for South Asia, New Delhi, Delhi, India; 2 Department of Biological Sciences and Biotechnology, Institute of Advanced Research, Gandhinagar, Gandhinagar, Gujarat, India; 3 International Maize and Wheat Improvement Centre, New Delhi, Delhi, India; 4 Department of Biotechnology, Thapar Institute of Engineering and Technology, Patiala, Punjab, India; 5 The Climate Corporation, Bayer Crop Science, Creve Coeur, MO, USA; 6 Global Wheat Program, International Maize and Wheat Improvement Centre, Texcoco, Mexico; 7 Department of Plant Pathology, Kansas State University, Manhattan, KS, United States of America

**Keywords:** Wheat, Genotyping-by-sequencing (GBS), SNP, Genetic diversity, Population structure, Analysis of molecular variance (AMOVA)

## Abstract

Genetic diversity and population structure information are crucial for enhancing traits of interest and the development of superlative varieties for commercialization. The present study elucidated the population structure and genetic diversity of 141 advanced wheat breeding lines using single nucleotide polymorphism markers. A total of 14,563 high-quality identified genotyping-by-sequencing (GBS) markers were distributed covering 13.9 GB wheat genome, with a minimum of 1,026 SNPs on the homoeologous group four and a maximum of 2,838 SNPs on group seven. The average minor allele frequency was found 0.233, although the average polymorphism information content (PIC) and heterozygosity were 0.201 and 0.015, respectively. Principal component analyses (PCA) and population structure identified two major groups (sub-populations) based on SNPs information. The results indicated a substantial gene flow/exchange with many migrants (Nm = 86.428) and a considerable genetic diversity (number of different alleles, Na = 1.977; the number of effective alleles, Ne = 1.519; and Shannon’s information index, *I* = 0.477) within the population, illustrating a good source for wheat improvement. The average PIC of 0.201 demonstrates moderate genetic diversity of the present evaluated advanced breeding panel. Analysis of molecular variance (AMOVA) detected 1% and 99% variance between and within subgroups. It is indicative of excessive gene traffic (less genetic differentiation) among the populations. These conclusions deliver important information with the potential to contribute new beneficial alleles using genome-wide association studies (GWAS) and marker-assisted selection to enhance genetic gain in South Asian wheat breeding programs.

## Introduction

Bread wheat (*Triticum aestivum* L.) is an allohexaploid species originating from successive rounds of hybridization in the Fertile Crescent during the Neolithic time, ∼8,000 to 10,000 years ago (Sansaloni et al., 2020). Wheat is the largest contributor to world grain production, with nearly 30% of total grain production and 50% of the world grain trade (Akter & Rafiqul Islam, 2017). Wheat grain is among the most consumed grains worldwide, providing 15% of calories every day, covering more than 220 million hectares with almost 750 metric megatons production every year (Balfourier et al., 2019). The global demand for wheat has increased due to population growth, changing food consumption habits, and socio-economic environments, specifically in African and Asian countries (Mondal et al., 2016).

During the domestication process, a substantial loss of diversity resulted in genetic bottlenecks. Researchers have been interested in using the genetic diversity of *Triticeae* species. Some of the vital gene pools include *Agropyron, Aegilops, Elymus, Leymus, Hordeum, Secale, Triticum,* and *Thinopyrum*. Above mentioned rich gene pools could improve various traits such as tolerance to biotic and abiotic stresses and micronutrient availability. Novel alleles from around fifty-two species have been introgressed, pointing out the significance of exotic introgressions in the breeding (Wulft & Moscou, 2014).

Population structure, genetic diversity, and relationships among genotypes are vital in scheming appropriate breeding plans (Peterson et al., 2014; Singh, Agarwal & Yadav, 2019). The population structure information aids in estimating the accurate association between phenotypic and genotypic variation (Knowler et al., 1988). The population structure information allows researchers to utilize natural diversity to detect vital genes/QTLs using current genetic technologies (Zhu et al., 2008). Genetic diversity studies have advanced from mere detection of distinct morphological to molecular traits investigations of DNA variation (Zhang, Maroof & Kleinhofs, 1993). Determining the current genetic diversity of crops has paramount importance for the selection and conservation of parents with the various genetic framework, thus providing well-organized crop enhancement (Mengistu, Kiros & Pè, 2015).

The first generation of morphological markers could not present the actual picture of diversity because of their limited number, lack of information about environmental and epistatic interaction. Slowly the paradigm shifted to investigate genetic diversity and population structure using PCR (polymerase chain reaction) based markers, which gave a better view of underlying diversity markers due to their abundance and environmentally neutral nature (Farahani et al., 2019). Among various molecular markers, the SNP marker is the preferred molecular marker in several crops due to extensive genome coverage, chromosome-specific location, low cost, co-dominant inheritance, and fast-tracking compared to other PCR-based markers. SNP are evolutionarily stable due to lower rates of recurrent mutation. SNPs are considered first-rate to understand genomic evolution and to study complex traits. SNPs have been extensively used in genetic resource characterization, genome-wide association studies (GWAS), genomic selection (GS), and marker-assisted selection (MAS) (Ganal, Altmann & Röder, 2009).

Microarrays have been used as a pre-eminent answer to develop SNPs in polyploid wheat genomes (Wang et al., 2014). Once an all-inclusive SNP data is accessible for species, a cost-effective microarray may be formed, and the process is relatively convenient. Microarray evades the miscalling risk of diversity on homoeologous genomes and lately with amplified 100 fold in wheat moving from 9 K (Cavanagh et al., 2013) to 820 K (Winfield et al., 2016). The 90 K wheat SNP array (Wang et al., 2014) has been effectively utilized for genetic diversity investigation, the building of high-density maps, and GWAS (Maccaferri et al., 2015; Chen et al., 2016; Mengistu et al., 2016; Wen et al., 2017). The low cost of genotyping by sequencing (GBS) makes it a robust approach for discovering and genotyping SNPs in various crops. GBS has been efficiently applied in genomic diversity, genetic linkage studies, and genomic selection in largescale plant breeding programs. GBS is found to be a perfect platform from single-gene to whole-genome sequencing and suited to genetic analysis and marker development (Heffner, Sorrells & Jannink, 2009; Heffner et al., 2010; Jannink, Lorenz & Iwata, 2010; Fu & Peterson, 2011; Poland & Rife, 2012; Poland et al., 2012; Thomson et al., 2012; Lu et al., 2013; Fu, Cheng & Peterson, 2014). The GBS has been established with maize and barley inbred populations with roughly 200,000 and 25,000 sequence tags, respectively (Elshire et al., 2011).

In the current study, GBS was used for genotyping 141 elite advanced breeding lines of spring wheat from CIMMYT (Mexico). The objectives were to illustrate the population structure and genetic diversity within and among subgroups. The present study not only defines the population structure and genetic diversity in these elite advanced wheat breeding lines also places a groundwork for genome-wide association study in this panel.

## Materials and Methods

### Plant genetic material

One hundred forty-one advanced breeding lines (ABLs) developed at CIMMYT, Mexico using adapted cultivars from various worldwide breeding programs were used in studying genetic diversity. These lines have been selected from a more extensive set of advanced breeding lines sent to South Asia as part of the CIMMYT wheat breeding program to develop high-yielding varieties suitable for the region. The lines were introduced in 2016 as the 4th cohort of the South Asia Bread Wheat Genomic Prediction Yield Trial (SABWGPYT). The lines were developed by making crosses with diverse and high yielding parental lines as part of the CIMMYT Global Wheat Program (GWP). A set of approx. thirty-eight thousand lines were planted in small plots in Obregon during the 2015-16 crop season. The selection was made based on yield and agronomic traits. Then lines from different genetic backgrounds were carefully selected as candidate varieties suitable for further testing in South Asia. The pedigree details data of those 141 advanced breeding lines are given in Table S1.

### Genotyping-by-sequencing and SNP filtering

Genomic DNA was extracted using the modified CTAB method from the fresh leaves of wheat seedlings (Dreisigacker et al., 2016). Genotyping-by-sequencing was performed in Illumina HiSeq 2500 using protocol from Poland et al. (2012). SNP calling was performed using TASSEL v5.2.6 (Bradbury et al., 2007) using the TASSEL-GBSv2 pipeline and aligned to the reference Chinese Spring Wheat Assembly (RefSeq v1.0). Beagle v4.1 with default settings was used to impute missing data. After filter criteria quality control (sample call rate >0.8, MAF ≥ 0.05, SNP call rate >0.7), 14,563 polymorphic SNPs and 141 genotypes were selected for further analysis.

### Genetic analysis of SNPs

The genotypic summary of the 14,563 SNPs was obtained using the “geno summary” function of TASSEL v5.2.6 (Bradbury et al., 2007). Chromosome-wise genomic SNP distribution, minor allele frequency (MAF), observed heterozygosity, and the polymorphism information content (PIC) were performed using GBS-based SNP markers. The PIC value of each biallelic SNP marker was computed using the following method derived from (Botstein et al., 1980):

\begin{eqnarray*}PIC=1-(MA{F}^{2}+{ \left( 1-MAF \right) }^{2})-(2MA{F}^{2}{ \left( 1-MAF \right) }^{2}) \end{eqnarray*}

### Population structure analysis

The population structure of the advanced bread wheat breeding lines was inferred using the Bayesian method implemented in STRUCTURE 2.3.4 (Pritchard, Stephens & Donnelly, 2000). Ten individualistic evaluations of every k were used. The STRUCTURE was placed on 100,000 burn-in, subsequently 100,000 Markov chain Monte Carlo (MCMC) replications. The best k for the present population was determined using the evanno algorithm (Evanno, Regnaut & Goudet, 2005) implemented in Pophelper v2.3.1 (Francis, 2017). Optimal K/sub-population was identified by the delta k-value peaks across various k values. Principal component analysis (PCA) based on covariates was performed of the SNP data in Tassel 5.2.6 (Bradbury et al., 2007) and plotted using Plotly v4.9.3 (Sievert, 2020). The PCAs were used to construct dendrograms using r package ape v5.4-1 (Paradis & Schliep, 2019) using the complete linkage method for hierarchical clustering.

### Genetic diversity analysis and analysis of molecular variance (AMOVA)

The selected K value from structure output was used to subdivide the advanced wheat lines into sub-populations and was utilized for AMOVA. AMOVA and genetic diversity indices were performed for individual sub-population using GeneAlEx v6.41 (Peakall & Smouse, 2006). The percentage of molecular variance among and within subgroups was calculated from AMOVA. The haploid number of migrants (Nm) was calculated using the among-population variance (Va) and within-population variance (Vw) using the formula

\begin{eqnarray*}{N}_{m}= \left[ \left\{ 1 \left/ \right. \left( \frac{{V}_{a}}{{V}_{a}+{V}_{w}} \right) \right\} -1 \right] /2 \end{eqnarray*}

The calculation of Shannon’s Information Index (*I*), effective alleles (Ne), different alleles (Na), number of loci with private alleles, unbiased diversity (uh), and the haploid genetic diversity index (h) were also performed.

Na was calculated by direct count of alleles across subpopulations per loci and averaged by the arithmetic mean across loci per sub-population. Ne was calculated using expected heterozygosity by formula

\begin{eqnarray*}Ne= \frac{1}{1-He} \text{where}~He=1-\sum {p}^{2} \end{eqnarray*}

Here p is the frequency of the allele. *I* was calculated (per locus and averaged across the number of loci) using the formula.

\begin{eqnarray*}I=-\sum {p}_{i}~ln~{p}_{i} \end{eqnarray*}

Where ln is the natural logarithm of p_i,_ i.e., frequency of ith allele, private alleles are the alleles unique to the subpopulation. The haploid genetic diversity index (h) provides the probability that the two individuals would be different and was calculated using the formula.

\begin{eqnarray*}h=1-\sum p_{i}^{2} \end{eqnarray*}

The unbiased diversity (uh) was calculated using the allele frequency and sample size (n) with a formula.

\begin{eqnarray*}uh= \frac{n}{n-1} \left( 1-\sum p_{i}^{2} \right) \end{eqnarray*}

## Results

### SNPs distribution on the wheat genome

A total of 14,563 bi-allelic SNPs were equitably distributed across the three genomes. The SNP HapMap and SNP positions datasets correspond to the reference Chinese Spring Wheat Assembly (RefSeq v1.0) used for present analysis have been provided through the link (https://doi.org/10.6084/m9.figshare.14273339.v2.). The Chinese Spring Wheat Assembly (RefSeq v1.0) reference genome assembly used in the present study is a widely used reference genome in hexaploid wheat. (https://urgi.versailles.inra.fr/download/iwgsc/IWGSC_RefSeq_Assemblies/v1.0/ and https://urgi.versailles.inra.fr/download/iwgsc/IWGSC_RefSeq_Annotations/v1.0/). The B-genome had a maximum of 7378 SNPs (50.66%), subsequently the A-genome with 5921 SNPs (40.66%) and the D-genome with a minimum number of SNPs, i.e., 1264 (8.68%) (Table 1, Fig. 1A). With 14,563 markers across the genome and genomic coverage of 13.9 GB, the average marker density was one marker per 0.95 Mb. The highest marker density with one marker every 0.54 Mb was observed on chromosome 2B, and the lowest density with one marker per 6.854 Mb on chromosome 4D (Fig. S1). The SNPs per chromosome ranged from 74 (4D) to 1486 (2B). The minimum and maximum SNPs detected on individual chromosomes ranged from 574 (4A) to 1293 (7A), 378 (4B) to 1486 (2B), and 74 (4D) to 255 (1D) at A, B, and D genome. The homoeologous group 7 had a maximum number of SNPs, 2838, followed by group 2 with 2652 SNPs, and group 4 had a minimum number of 1026 SNPs.

**Table 1:** Chromosome wise distribution of SNP markers across the wheat genomes.

**Group**	**SNPs**	**First SNP**	**Last SNP**	**Coverage**	**Dist. b/w SNP**	**Density**
1A	686	1.145	593.790	592.645	0.864	1.158
1B	881	1.299	688.328	687.028	0.780	1.282
1D	255	0.079	493.979	493.900	1.937	0.516
2A	912	0.718	771.354	770.635	0.845	1.183
2B	1486	0.019	800.870	800.851	0.539	1.856
2D	254	8.791	649.074	640.282	2.521	0.397
3A	832	0.608	750.539	749.931	0.901	1.109
3B	1027	0.580	829.535	828.955	0.807	1.239
3D	115	24.795	615.062	590.267	5.133	0.195
4A	574	2.705	744.515	741.810	1.292	0.774
4B	378	1.056	666.052	664.996	1.759	0.568
4D	74	3.238	509.798	506.560	6.845	0.146
5A	799	1.294	709.755	708.461	0.887	1.128
5B	1084	8.072	712.941	704.869	0.650	1.538
5D	106	62.140	555.015	492.875	4.650	0.215
6A	825	0.684	617.839	617.155	0.748	1.337
6B	1227	0.196	720.805	720.609	0.587	1.703
6D	210	0.070	473.287	473.217	2.253	0.444
7A	1293	2.083	736.572	734.489	0.568	1.760
7B	1295	1.021	750.125	749.105	0.578	1.729
7D	250	2.145	635.422	633.277	2.533	0.395
Genome A	5921			4915.126	0.830	0.090
Genome B	7378			5156.413	0.699	0.057
Genome D	1264			3830.378	3.030	0.030
**Total**	14563			13901.92	1.048	0.955

**Notes.**

# Position and coverage of SNPs is in megabases (Mb), Distances between SNPs in Mb, Density = No of SNPs per MB

**Figure 1:**
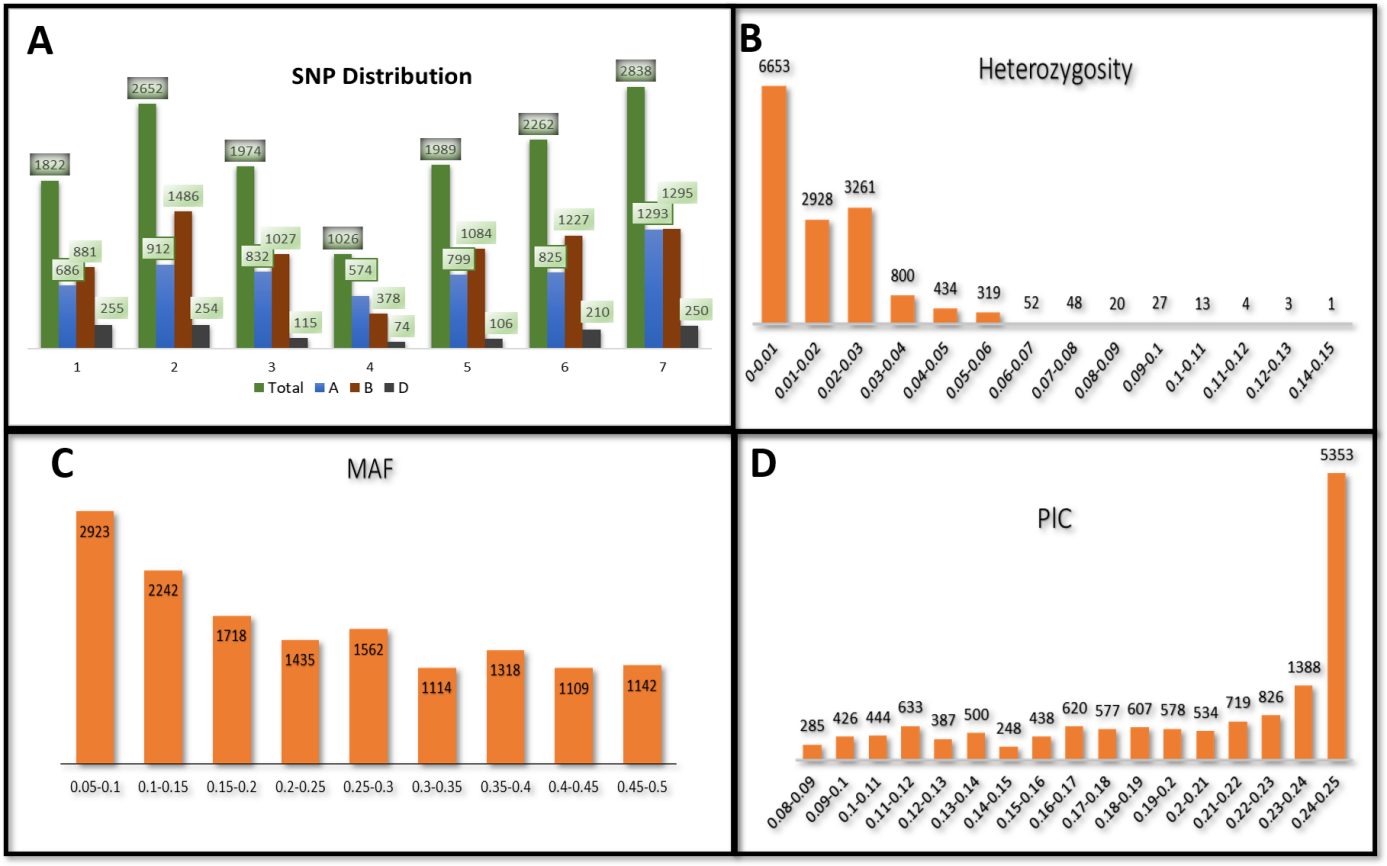
Distribution of 14,563 SNPs based on chromosomal distribution and markers characteristics. (A) Distribution of SNP markers across 21 chromosomes of bread wheat for 141 advanced wheat lines, (B) distribution of the percentage of heterozygosity (He), (C) distribution of the minor allele frequency (MAF), and (D) distribution of the polymorphic information content (PIC).

### Genetic analysis of SNPs

The heterozygosity among all SNPs ranged from 0.00 to 0.149. Twenty-one SNPs showed high heterozygosity (>0.10), and 3,095 SNPs showed zero heterozygosity (Fig. 1B, Table S2). The heterozygosity was less than 0.05 for around 97% of the SNPs. SNPs in chr7A showed the highest heterozygosity, with SNP S7A_717968190 having heterozygosity of 0.149. The three ABLs, namely GID7396143, GID7399653, and GID7400318, were highly heterologous with heterozygosity equal to 0.16, 0.15, and 0.12, respectively (Table S3). The MAF ranged from 0.05 to 0.50 in the present study. Fifty-five SNPs were found with MAF =0.50 (where both the alleles of these SNPs were equally distributed across the panel). The average observed MAF was 0.23, while most ranged from 0.05 to 0.10, i.e., 2923 SNPs (Fig. 1C). The PIC values ranged from 0.085 to 0.250, with 54 SNPs having a PIC value equal to 0.250 (Fig. 1D). 5353 SNPs (36%) showed a range of 0.24−0.25 for PIC and were the highest among the SNPs.

### Population structure analysis

**Figure 2:**
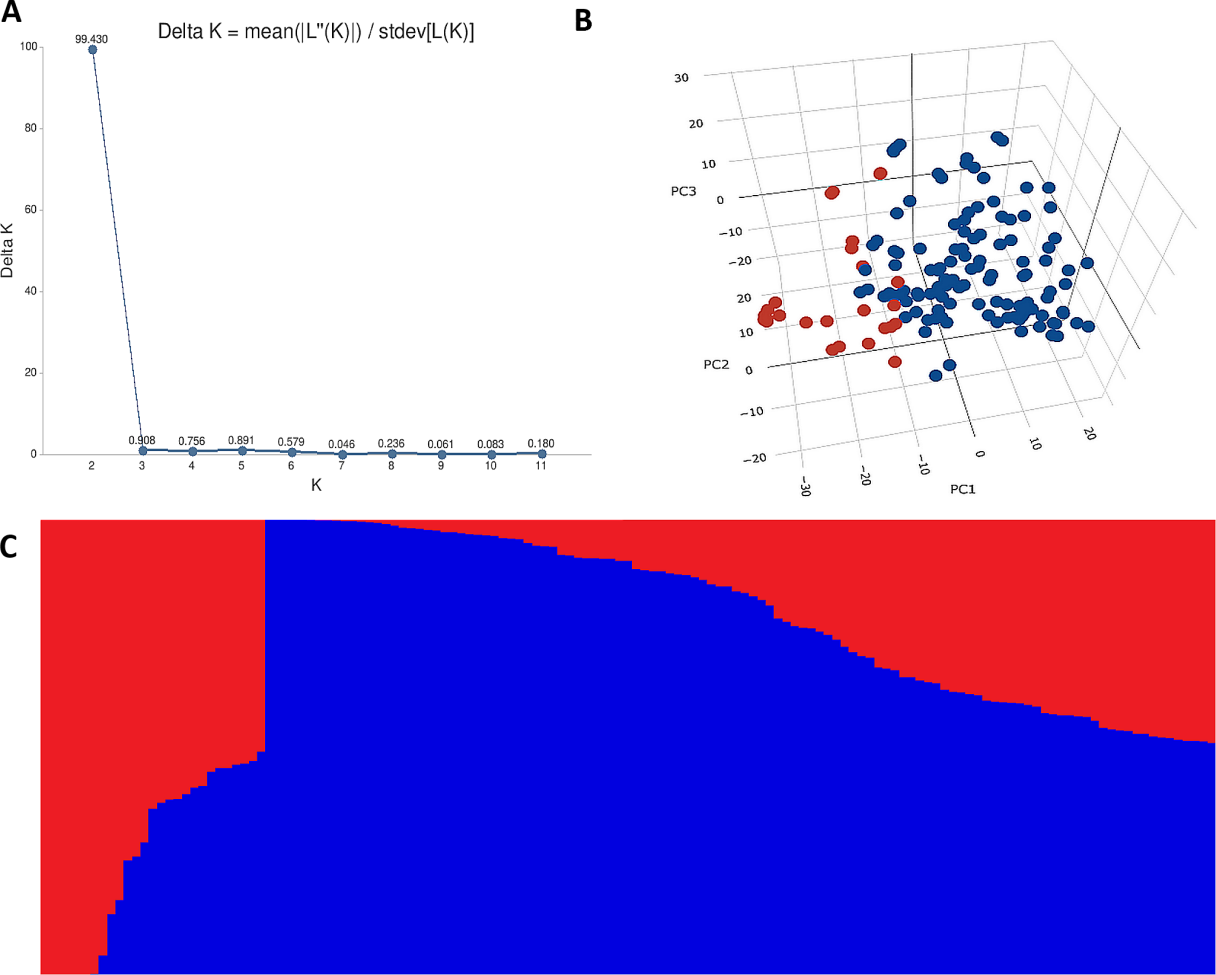
Population structure analysis of 141 advanced bread wheat lines. STRUCTURE results based on 14,563 GBS markers. (A) Sharp peak was observed at *K* = 2 with a maximum of ΔK determined by the Evanno method showing the stratification of the population into the minimum number of possible subgroups in the advanced wheat breeding lines. (B) Scatter plot of three principal components (PCs) of the SNP data with lines colored according to the two subgroups. (C) Structure plot for 141 advanced wheat breeding lines was stratified into two distinct clusters, where each color represents one subgroup representing (G1) and (G2).

**Figure 3:**
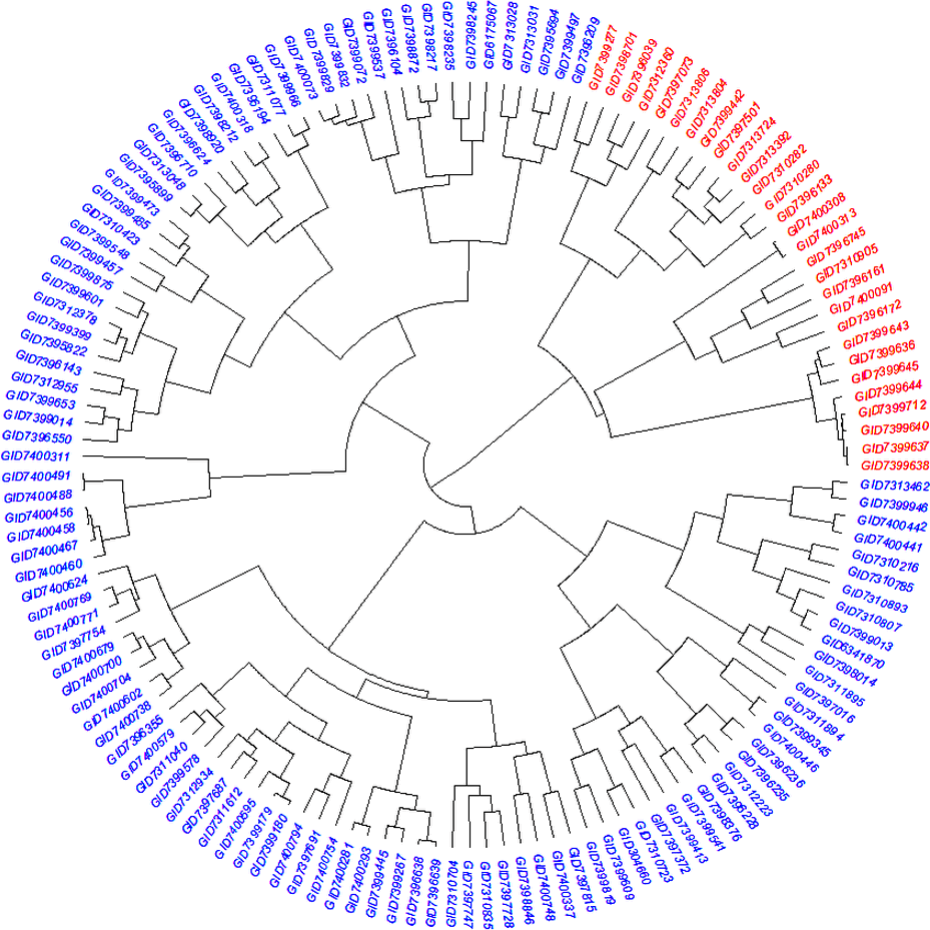
Dendrogram demonstrating the genetic relationships among 141 advanced bread wheat lines based on 14,563 GBS markers. Dendrogram showing the relationship among advanced breeding lines. Phylogenetic network constructed using complete linkage hieratical clustering based on 14,563 SNPs for all 141 advanced breeding lines. Breeding lines labels were color-coded into two subgroups.

The STRUCTURE results for the 141 ABLs showed that ΔK was highest at *K* = 2, indicating the presence of two major subgroups (Fig. 2A). The output of the STRUCTURE for *K* = 2 is given in Fig. 2B. The structure out showed very high ΔK for *K* = 2, compared to other K values, only a few genotypes represented complete distinction between the subgroups. Some genotypes across the subgroups showed near equal distribution of alternative alleles. Similar results were revealed by the PCA analysis where the genotypes of the subgroups could be seen clustered near each other when the first three principal components were observed (Fig. 2C). The dendrogram based on PCA also showed similar results (Fig. 3).

### Analysis of molecular variance (AMOVA)

**Figure 4:**
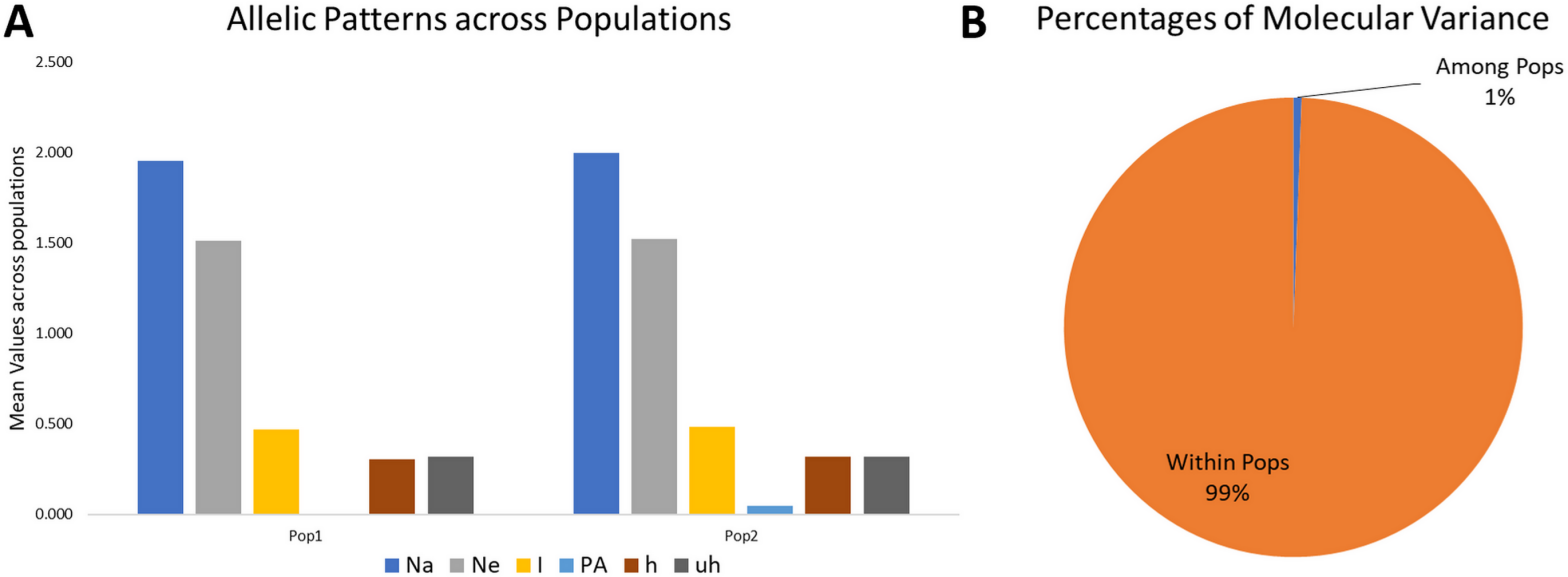
Proportion of Allelic patterns and diversity. (A) The allelic patterns and diversity indexes for *K* = 2, each subpopulation based on number of different alleles (Na), number of effective alleles (Ne), Shannon’s information index(*I*), private alleles (PA), and diversity index (h) and (B) diversity among and within subgroups for *K* = 2.

The analysis was performed considering *K* = 2. The variance among groups for *K* = 2 explained only 1% of the total variance and 99% variance within the subgroups. It indicates that the genetic differentiation within subgroups was high. Simultaneously, it was low among the subgroups (Table 2, Fig. 4), which may be due to the low genetic differentiation or excessive gene traffic between the population set. The high gene traffic is supported by the variation trends of both percentages of variance explained and haploid Nm values. The Nm values (haploid number of migrants) was very high, with Nm = 86.428.

### Allelic pattern covering the populations

The mean Shannon’s information index (I), the diversity index (h), and the unbiased diversity index (uh) were high (0.477, 0.313, and 0.320), which indicated its high diversity (Table 3, Fig. 4). The mean of different alleles (Na) and the effective alleles (Ne) of the *K* = 2 population set were 1.977 and 1.519. The different alleles (Na) in the subgroup 1 (G1) and subgroup 2 (G2) were 1.954 and 2.0, respectively, indicating comparatively better diversity in G2 than G1 (Table 3).

**Table 2:** Analysis of Molecular Variance for *K* = 2 for within population and among-population variation.

**K**	**Source**	**df**	**SS**	**MS**	**Est. Var.**	**%**	**Nm**
**2**	Among Pops	1	3094.516	3094.516	14.292	1%	86.428
	Within Pops	139	343401.995	2470.518	2470.518	99%	
	Total	140	346496.511		2484.810	100%	

**Notes.**

#K = K value for sub-populations, Source = source of variation, df = degree of freedom, SS = sum of squares, MS = mean sum of squares, Est. Var.= estimated variation, % = percentage of variance explained, and Nm = haploid number of migrants.

**Table 3:** Allelic patterns and diversity indexes for *K* = 2, for subpopulations and mean diversity among subpopulations for each K.

**Diversity indexes**	**Pop1**	**Pop2**	**Mean**
Na	1.954 (±0.002)	2.000 (±0.000)	1.977
Na Freq. ≥ 5%	1.870 (±0.003)	1.973 (±0.001)	1.921
Ne	1.512 (±0.003)	1.525 (±0.002)	1.519
I	0.468 (±0.002)	0.487 (±0.001)	0.477
h	0.307 (±0.001)	0.318 (±0.001)	0.313
uh	0.320 (±0.001)	0.321 (±0.001)	0.320
PA	6.8E−5 (±0.000)	0.046 (±0.002)	0.023
Lines with PA	22	90	
Range of PA	3–149	3–188	

**Notes.**

# Na = number of different alleles, Ne= number of effective alleles, I = Shannon’s information index, PA = private alleles, h = diversity index, and uh = unbiased diversity index, ± represents standard error

Similarly, the numbers of effective alleles (Ne) was relatively higher in G2 (1.525) than in G1 (1.512). However, only G2 showed a significant percentage of private alleles, i.e., 4.6%. G1 contained 22 members with private allele ranging from 7 in GID7399640 to 149 in GID7400491, while G2 contained 90 members with private alleles ranging from 3 in GID7396143 to 188 in GID7400293 (Table 3 & Table S4). The diversity indexes *I*, h and uh for G1 & G2 were 0.468 and 0.487, 0.307 and 0.318, 0.320 and 0.321, respectively, indicating relatively higher diversity within G2 than G1.

## Discussion

A panel of 141 genotypes from an extensive collection of elite advanced bread wheat breeding lines from CIMMYT, Mexico, was used to study the genetic diversity (Table S1). The high-throughput SNP genotypic data obtained through GBS was used to explore population genetics and genetic diversity, supporting future breeding efforts (e.g., GWAS) in the bread wheat breeding program in South Asia.

The majority of the 14,563 SNPs were distributed on A and B genome, and only 8.67% SNPs were on D genome, which is at par with earlier findings (Chao et al., 2009; Akhunov et al., 2010; Berkman et al., 2013; Würschum et al., 2013; Marcussen et al., 2014; Shavrukov et al., 2014; Edae, Bowden & Poland, 2015; Alipour et al., 2017; Eltaher et al., 2018; Rufo et al., 2019). The lower SNPs across the D genome indicates its young wheat evolutionary past and less genetic diversity (Caldwell et al., 2004; Alipour et al., 2017; Eltaher et al., 2018), which could be explained by lower recombination rates and frequency in the D genome (Chao et al., 2009). This could further be defined as a larger wild emmer diversity which contributed to hexaploid formation than *Ae. tauschii* (D-genome donor) (Dubcovsky & Dvorak, 2007). Significant initial gene movement must have occurred amongst *T. aestivum* and *T. turgidum* (AABB); however, not amongst *Ae. tauschii* (DD) and hexaploid (Caldwell et al., 2004; Dvorak et al., 2006). It leads to less genetic diversity in the D genome compared to the A and B genomes (Talbert, Smith & Blake, 1998; Caldwell et al., 2004; Dvorak et al., 2006; Berkman et al., 2013). The role of A, B, and D genomes to genetic diversity of hexaploid wheat were reported prior via diverse markers systems, i.e., RFLPs, AFLP, SSRs, DArT (Liu & Tsunewaki, 1991; Röder et al., 1998; Peng et al., 2000; Nielsen et al., 2014). The smallest number of SNP were on 4D, while the maximum markers were positioned on chromosome 2B, which agrees with Allen et al. (2017) and Bhatta et al. (2017). Previous studies also reported the lowest number of SNPs at the 4D (Saintenac et al., 2013; Sukumaran et al., 2014; Allen et al., 2017; Alipour et al., 2017; Bhatta et al., 2017). While the highest SNP have been on a different chromosome in some of these studies, i.e., 3B (Saintenac et al., 2013; Alipour et al., 2017) and 1B (Sukumaran et al., 2014).

The low levels of observed heterozygosity (0.00−0.149), with approximately 97% of SNPs having heterozygosity <0.05 and only 21 SNPs having heterozygosity above 0.10, showed the panel had high genetic stability (Kristensen et al., 2018; Rimbert et al., 2018; Chu et al., 2020; Sun et al., 2020). Since these lines would/may not segregate further across generation leading to stable phenotypic evaluations. The lines with the low heterozygosity are highly desirables for selection as parental genotypes in any breeding program. Furthermore, the least heterozygous lines (GID7395694, GID7399636, GID7399643, GID7399645, and GID7400337) and highly heterozygous lines (GID7396143, GID7399653, and GID7400318) were observed among ABLs (Table S3). High heterozygosity among these three ABLs indicates either genomic instability or the higher outcrossing ability of these lines. MAF could easily arbitrate the allelic distribution of the SNPs. In this study, 55 SNPs were identified with MAF equal to 0.50. Traits showing distribution patterns similar to these SNPs could be easily associated with such SNPs.

PIC values are signals of informative markers in the crops and reflect through the spreading of informative markers in the genome, which can be used for studying genetic diversity (Nielsen et al., 2014; Salem & Sallam, 2016). The moderately informative PIC values indicate the SNPs’ bi-allelic nature, which is limited to PIC 0.5, where both the alleles have similar occurrences (Eltaher et al., 2018). Another reason is the slow nucleotide mutation rate in GBS-SNPs compared to the mutation rate of SSRs (Thuillet et al., 2002; Chesnokov & Artemyeva, 2015). Most of the SNPs were moderately informative, with PIC values ranged from 0.085 to 0.250. The average PIC value among all sites was 0.201 (Fig. 1D). Lopes et al. (2015) used the 9K SNP for the WAMI population, detected a PIC value of 0.27 and disclosed that spring wheat confined moderate polymorphism. In another study, Novoselović et al. (2016) obtained an average moderate polymorphism of 0.30 PIC amongst the Croatian population by 1,229 Diversity Arrays Technology (DArT) markers. Furthermore, El-Esawi et al. (2018) also found moderate PIC (0.33 and 0.29) in Australian and Belgian wheat, respectively. Intrestingly, Eltaher et al. (2018) also detected a moderate PIC value of 0.25 in 270 F3V6 Nebraska winter wheat. The present study outcome is following the above-mentioned previous studies.

In the present study, the STRUCTURE analysis was identified by ΔK with the highest peak at *K* = 2, which is vital for the elucidation of genetic diversity. Winfield et al. (2016) used 32,443 SNP markers and 804 wheat genotypes collected from over 30 countries. They detected that most European wheat accessions were grouped together, divided from the Asian and Middle Eastern accessions. Cavanagh et al. (2013) also reported that the winter wheat from the European population displayed robust genetic differentiation in their study. Chen et al. (2019) described that West Asian, European, numerous Central and South Asian landraces, and most East Asian cultivars grouped in the same cluster, whiles most of the East Asian landraces were grouped with South, Central and West Asian landraces. Lee et al. (2018) described that most Japanese, Korean and genotypes from Afghanistan were grouped in a cluster, while the Middle Eastern, Chinese, and Caucasus germplasm were in a separate group. The genetic diversity and population structure in the current ABLs were not surprising since the genetic composition, despite being variable, is restricted due to common ancestry (Table S1), leading to closely linked clusters. PCA and dendrogram results were in agreement with STRUCTURE results. They exhibited closely related groups, which might be because the selection of lines was based on traits in wheat, e.g., yield, biotic, and abiotic resistance arising from the parental pedigree of genotypes. Besides genetic diversity analysis, genetic structure analysis for subgroups composition is also an essential part of genome-wide association studies (GWAS) to counter false positives arising due to common ancestry among the panel of genotypes (Yao et al., 2019). Hence, population interchange and exploitation of global germplasm have become an essential preliminary step to increase the genetic source for wheat breeding (Zhao et al., 2019).

Private alleles provide important information identifying distinctive genetic variability at loci and diversified genotypes, which could be employed in crop breeding to enhance the allele affluence in a population (Borba et al., 2009; Salem & Sallam, 2016). For *K* = 2, G1 and G2 contained 22 and 90 members with private alleles, respectively, illustrating a clear difference in the lines containing private alleles (Table S4). These results indicate G2 being genetically diverse compared with subgroup G1, further supported by slightly higher values of diversity indexes in G2 (0.318) than G1(0.307). Similar results have been previously observed in studies on wheat genotypes using SNP markers where higher values of *I, u* and *uh* in a subgroup are indicative of a higher diversity of the group (Alipour et al., 2017; Eltaher et al., 2018; Kumar et al., 2020; Mourad, Belamkar & Baenziger, 2020). A system should be designed to identify private alleles equipped for receiving and harnessing the essential adaptive genes.

The AMOVA results showed high genetic diversity within-subgroups; however, the diversity between subgroups was very low (1%). The result may be due to the common parental backgrounds and selection based on designated agronomic traits resulted in high gene flow levels. This low level of variation among the stratified groups occurs due to increased gene exchange described by Arora et al. (2014). The allelic outlines elucidated valuable evidence on genetic diversity in each subgroup. Wright (1965) also described restricted Nm (haploid) gene flow between populations. In the current study, a very high Nm value (86.428), suggesting a high level of genetic exchange/flow among the subgroups, caused small genetic variation (Eltaher et al., 2018). Hence, the high genetic interchange amid subgroups directed to a small genetic variation amongst subgroups. The variation amongst subgroups was noteworthy (*P* <0.001) regardless of being low (1%). The present study results will aid breeders to understand the genetic diversity and perform marker-assisted selection on this panel.

### Conclusion

In the present study, we applied GBS-based SNP to learn GBS-SNP markers’ usefulness for diversity analysis in 141 elite wheat breeding lines. Despite very designated, our advanced breeding lines panel was found to be genetically diverse, which could be instrumental for future South Asian breeding programs to develop new elite wheat varieties of alluring traits, i.e., high yield, biotic and abiotic resistance. Besides, the present study dappled two subgroups that were enlightened by their parentage and selection history. The low heterozygosity detected among elite advanced wheat breeding lines within subgroups and the moderate divergence among subgroups suggested that the elite advanced wheat breeding lines could be used further for GWAS studies.

##  Supplemental Information

10.7717/peerj.11593/supp-1 Supplemental Information 1 Pedigree information of advanced breeding lines

10.7717/peerj.11593/supp-2 Supplemental Information 2 SNP-wise major and minor allele frequency, heterozygosity, and polymorphism information content for the GBS based SNPs used in this study

10.7717/peerj.11593/supp-3 Supplemental Information 3 Genotype-wise heterozygosity of the SNPs accounting for the genotypic heterozygosity of the advanced breed wheat lines used in this study

10.7717/peerj.11593/supp-4 Supplemental Information 4 Details of private alleles of different lines within subgroups Pop1 is subgroup1, pop2 is subgroup2, column D contains the names of private alleles in respective lines.

10.7717/peerj.11593/supp-5 Supplemental Information 5 Distributions of GBS based SNP markers across the 21 chromosomes of wheat genome

10.7717/peerj.11593/supp-6 Supplemental Information 6 Supplemental GBS HapMap

## Additional Information and Declarations

### Data Availability

The following information was supplied regarding data availability: The GBS SNP Data is available in the Supplemental File. The SNP dataset used in the present study is also available at: Tomar, Vipin (2021): WheatGBS.hmp.txt. figshare. Dataset. https://doi.org/10.6084/m9.figshare.14273339.v2. SNPs were aligned and assigned to an exact physical location by referencing the Chinese Spring Wheat Assembly v1.0 (International Wheat Genome Sequencing Consortium [IWGSC]), and used for further downstream analysis.
