# Supplementary figures and images for: Elucidating SNP-based genetic diversity and population structure of advanced breeding lines of bread wheat (Triticum aestivum L.)
Source: PeerJ. 2021 Jun 22;9:e11593. doi: 10.7717/peerj.11593 (PMC8231316; doi:10.7717/peerj.11593)

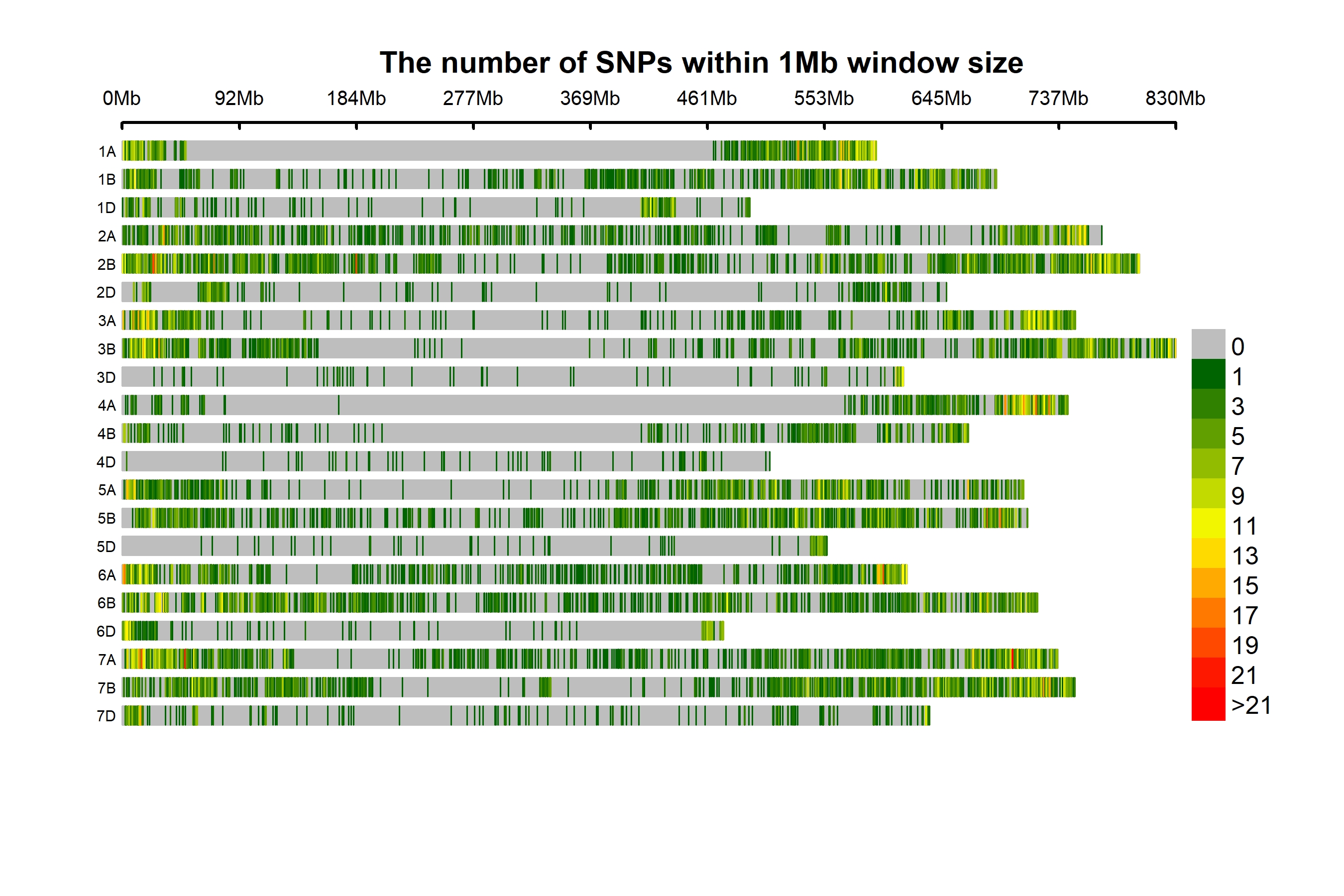

Supplement: Supplemental Information 5 [file peerj-09-11593-s005.jpg]
